# Morphology and morphogenesis of SARS-CoV-2 in Vero-E6 cells

**DOI:** 10.1590/0074-02760200443

**Published:** 2021-02-08

**Authors:** Debora Ferreira Barreto-Vieira, Marcos Alexandre Nunes da Silva, Cristiana Couto Garcia, Milene Dias Miranda, Aline da Rocha Matos, Braulia Costa Caetano, Paola Cristina Resende, Fernando Couto Motta, Marilda Mendonça Siqueira, Wendell Girard-Dias, Bráulio Soares Archanjo, Ortrud Monika Barth

**Affiliations:** 1 Fundação Oswaldo Cruz-Fiocruz, Instituto Oswaldo Cruz, Laboratório de Morfologia e Morfogênese Viral, Rio de Janeiro, RJ, Brasil; 2 Fundação Oswaldo Cruz-Fiocruz, Instituto Oswaldo Cruz, Laboratório de Vírus Respiratórios e do Sarampo, Rio de Janeiro, RJ, Brasil; 3 Fundação Oswaldo Cruz-Fiocruz, Instituto Oswaldo Cruz, Plataforma de Microscopia Eletrônica Rudolph Barth, Rio de Janeiro, RJ, Brasil; 4 Instituto Nacional de Metrologia, Qualidade e Tecnologia, Núcleo de Laboratórios de Microscopia, Rio de Janeiro, RJ, Brasil

**Keywords:** SARS-CoV-2, Vero-E6 cells, morphology, morphogenesis

## Abstract

**BACKGROUND:**

The coronaviruses (CoVs) called the attention of the world for causing outbreaks of severe acute respiratory syndrome (SARS-CoV), in Asia in 2002-03, and respiratory disease in the Middle East (MERS-CoV), in 2012. In December 2019, yet again a new coronavirus (SARS-CoV-2) first identified in Wuhan, China, was associated with a severe respiratory infection, known today as COVID-19. This new virus quickly spread throughout China and 30 additional countries. As result, the World Health Organization (WHO) elevated the status of the COVID-19 outbreak from emergency of international concern to pandemic on March 11, 2020. The impact of COVID-19 on public health and economy fueled a worldwide race to approve therapeutic and prophylactic agents, but so far, there are no specific antiviral drugs or vaccines available. In current scenario, the development of *in vitro* systems for viral mass production and for testing antiviral and vaccine candidates proves to be an urgent matter.

**OBJECTIVE:**

The objective of this paper is study the biology of SARS-CoV-2 in Vero-E6 cells at the ultrastructural level.

**METHODS:**

In this study, we documented, by transmission electron microscopy and real-time reverse transcription polymerase chain reaction (RT-PCR), the infection of Vero-E6 cells with SARS-CoV-2 samples isolated from Brazilian patients.

**FINDINGS:**

The infected cells presented cytopathic effects and SARS-CoV-2 particles were observed attached to the cell surface and inside cytoplasmic vesicles. The entry of the virus into cells occurred through the endocytic pathway or by fusion of the viral envelope with the cell membrane. Assembled nucleocapsids were verified inside rough endoplasmic reticulum cisterns (RER). Viral maturation seemed to occur by budding of viral particles from the RER into smooth membrane vesicles.

**MAIN CONCLUSIONS:**

Therefore, the susceptibility of Vero-E6 cells to SARS-CoV-2 infection and the viral pathway inside the cells were demonstrated by ultrastructural analysis.

Coronavirus disease 2019 (COVID-19) presents a clinical spectrum ranging from asymptomatic individuals to more complex conditions such as severe acute respiratory syndrome. Although most patients have mild symptoms and good prognosis, an estimate 10-20% of individuals developed the severe forms of illness, and 2-5% may die due complications in multiple organs. The pathogenesis of the disease related to the severe acute respiratory syndrome of coronavirus 2 (SARS-CoV-2) in humans is still unclear.^1^ Among patients with pneumonia, fever was the most common symptom, followed by cough.^2^ Bilateral pulmonary involvement was the most common finding from chest computed tomography.^3^ Dissemination of SARS-CoV-2 occurs mainly by person-to-person transmission, through contact with respiratory fluids. It is estimated that the infection has an average incubation period of six days.

COVID-19 was first identified in Wuhan, Hubei Province, Republic of China, on December 1st, 2019, but the initial reports only came out on December 31st of the same year. Since the early stages of the epidemics, several evidences have pointed to a probable zoonotic origin for SARS-CoV-2, in particular, the initial observation that first cluster of infections was linked to a seafood and live animal wholesale market. Since its isolation from Wuhan samples in January 2020,^4^ SARV-CoV-2 has quickly spread in China and many other countries.^5^ ^,^ ^6^ ^,^ ^7^ ^,^ ^8^ ^,^ ^9^ ^,^ ^10^ On January 30th 2020, the World Health Organization (WHO) declared COVID-19 as the sixth public health emergency of international concern and, on March 11th 2020, raised the classification of SARS-CoV-2 outbreak to pandemic. By the time of the WHO announcement, more than 118,000 people had already been infected in 114 countries.^11^

SARS-CoV-2 is an enveloped, positive-sense RNA virus belonging to genus Betacoronavirus.^4^ ^,^ ^12^ ^,^ ^13^ Phylogenetic analysis revealed that SARS-CoV-2 is closely related (88-89% similarity) to SARS-like coronaviruses from bats, such as bat-SL-CoVZC45 (GenBank no. MG772933.1) and bat-SL-CoVZXC21 (GenBank no. MG772934.1), and shares lower similarity to SARS-CoV (~79% similarity) and MERS-CoV (~50% similarity).^4^ ^,^ ^14^ ^,^ ^15^ SARS-CoV-2 virions (infectious particles) have a diameter of approximately 50 to 200 nm. Like in other coronaviruses, the SARS-CoV-2 lipid envelope contains a spike protein (S), a membrane protein (M) and an envelope protein (E). The S protein mediates viral binding to the host cell membrane through interaction with the angiotensin conversion enzyme (ACE2) receptor.^16^ The nucleocapsid protein (N) forms the virion core, which encases the viral RNA genome.^7^ ^,^ ^17^ Currently there are no specific antiviral drugs or vaccines for treating and preventing COVID-19. There are, though, several candidates in different stages of development, with few recently reaching the phases of clinical testing. Standardised *in vitro* systems for viral mass production and infection modeling are essential tools to accelerate the initial steps of drug development, screening and pre-clinical testing. In this sense, several research groups have demonstrated the susceptibility of different cell lines to SARS-CoV-2 through molecular techniques.^18^ ^,^ ^19^ ^,^ ^20^ However, studies regarding the morphogenesis of SARS-CoV-2 in cell lineages are scarce in the literature so far. Here, we used a lineage of African green monkey kidney cells (Vero-E6) to isolate SARS-CoV-2 viruses from samples of nasopharyngeal swabs of patients positive for COVID-19. Using transmission electron microscopy, we were able to document the morphology and replication cycle of SARV-CoV-2, and the consequent ultrastructural alterations induced in the host cells.

## MATERIALS AND METHODS

*Clinical samples* - Nasopharyngeal swabs were collected from patients admitted to the sentinel health units of the national surveillance network for respiratory of the Brazilian Ministry of Health (MoH). Samples were obtained in different regions of the country and referred to the National Influenza Centre (NIC) at Fiocruz, Rio de Janeiro, for SARS-CoV-2 detection, as part of the COVID-19 surveillance program. Total RNA was extracted from clinical samples using the QIAmp Viral RNA mini kit (Qiagen). Viral detection was done by real time reverse transcription polymerase chain reaction (RT-PCR) with TaqMan primers and probes (IDT) specific for the genes encoding the Envelope protein (E) and the viral RNA-dependent RNA Polymerase (RdRp), as described previously.^21^ Reactions were performed with the Qiagen One Step RT-PCR kit (Qiagen, USA). Synthetic RNA sequences corresponding to E and RdRP targets^21^ were used as positive controls. Positive samples were then used for SARS-CoV-2 isolation in Vero-E6 cells. This research is approved by the Ethics Committee of Instituto Oswaldo Cruz (protocol number 2453470).

*Cells and viral isolation* - All cell culture reagents were acquired from Gibco. Prior to infection, Vero-E6 (African green monkey kidney) cultures were maintained in DMEM supplemented with 10% foetal bovine serum (FBS) and 100U/mL of penicillin-streptomycin (1x Pen-Strep) and cultured at 37ºC and 5% CO_2_ (Szretter et al.^22^). For infection, monolayers were washed twice with phosphate buffered saline (PBS) and inoculated with a clinical sample diluted in non-supplemented DMEM. Non-infected control cultures (mock) were prepared using pure non-supplemented DMEM as inoculum. After incubation for 1 h at 37ºC, the viral (and mock) inoculum was removed and cells were cultivated at 37ºC in DMEM supplemented with 2% FBS and 1x Pen-Strep. Monolayers were inspected daily under light microscope for development of cytopathic effect (CPE), until 72 h post infection (hpi). All procedures were performed in a biosafety level 3 laboratory, according to WHO guidelines. Whole-genome sequences of isolates evaluated in this study are available in the Global initiative on sharing all influenza data (GISAID) under the accession numbers EPI_ISL_415105, EPI_ISL_414045 e EPI_ISL_427294 (https://www.gisaid.org/).

*Viral quantification in cell cultures supernatants* - Viral quantities in cultures were estimated by determination of the number of copies of the viral gene E per volume (µL) of supernatant. Total RNA was extracted from culture supernatants using the QIAmp Viral RNA mini kit (Qiagen). Quantification of E gene copies was performed by real time RT-PCR using specific TaqMan primers and probes^21^ and the Qiagen one step RT-PCR kit (Qiagen, USA). A standard curve was set using a synthetic RNA control containing the sequence of E gene. The control, with initial concentration from 10^8^ copies/µL, was serially diluted (with factor 10) to obtain a series of 10^7^ copies/µL to 10 copies/µL.

*Transmission and high resolution scanning electron microscopies* - For analyses in electron transmission microscopy the infected and non-infected control (mock) monolayers were trypsinised at 24, 48 and 72 hpi. Cell suspensions were fixed in 2.5% glutaraldehyde in sodium cacodilate buffer (0.2 M, pH 7.2), post-fixed in 1% buffered osmium tetroxide, dehydrated in acetone, embedded in epoxy resin and polymerised at 60ºC over the course of three days.^23^ ^,^ ^24^ Ultrathin sections (50-70 nm) were obtained from the resin blocks. The sections were picked up using copper grids, stained with uranyl acetate and lead citrate,^25^ and observed using Jeol JEM 1011, FEI Titan, FEI Tecnai Spirit, Hitachi HT 7800 transmission electron microscopes. For analyses in high resolution scanning microscopy, infected and non-infected control (mock) monolayers were grown on sterile glass coverslips and fixed at 24, 48 and 72 hpi, in 2.5% glutaraldehyde in sodium cacodilate buffer (0.2 M, pH 7.2), dehydrated in ethanol and submitted to critical-point-dried. The cells were analysed in Orion NanoFab Helium Ion Microscope.

## RESULTS

*SARS-CoV-2 quantification from Vero-E6 cell culture supernatants* - To evaluate the ability of Vero-E6 cells to produce SARS-CoV-2 progeny, we quantified the number of copies of virus RNA in cell culture supernatants collected one and 72 hpi. The quantitative real time RT-PCR assay demonstrated an increase in the amount of SARS-CoV-2 RNA copies in the supernatant at least two log_10_ steps within 72 hpi (Table), suggesting production of viral progeny.

**TABLE Virus:** RNA quantification (copies/mL)

Virus isolate	1 hpi (inoculum)	72 hpi (virus growth)
EPI_ISL_415105	1,2 x 10^4^	1,3 x 10^7^
EPI_ISL_414045	1,3 x 10^5^	1,4 x 10^7^
EPI_ISL_427294	4,7 x 10^3^	1,6 x 10^7^

hpi: hours post infection.

*Morphological analysis of Vero-E6 cell cultures infected with SARS-CoV-2* - Analysis of cultures under inverted light microscopy demonstrated CPE in infected Vero-E6, which was mostly evident from 48 hpi. The CPE appeared as rounding and detaching of cells and formation of syncytia (data not shown).

**Fig. 1:**
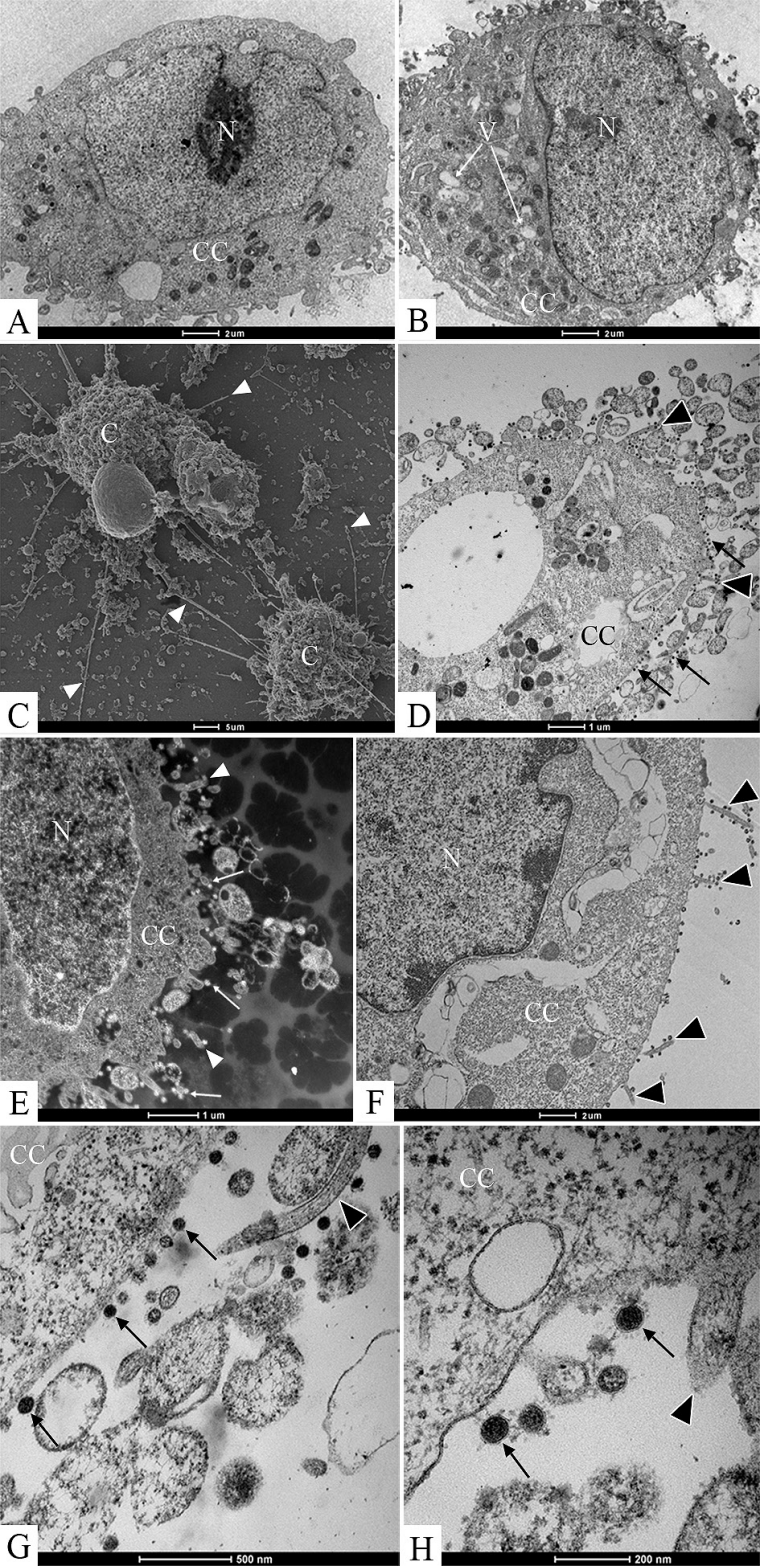
ultrastructural analyses of Vero-E6 cells by electron microscopy. (A) Uninfected cell presenting no morphological alterations. (B-H) Vero-E6 cell, 72 h post infection (hpi) with severe acute respiratory syndrome (SARS-CoV-2), presenting numerous filopodia (head arrow) and vesicles (V). SARS-CoV-2 particles (arrow), nucleus (N), cell cytoplasm (CC).

**Fig. 2:**
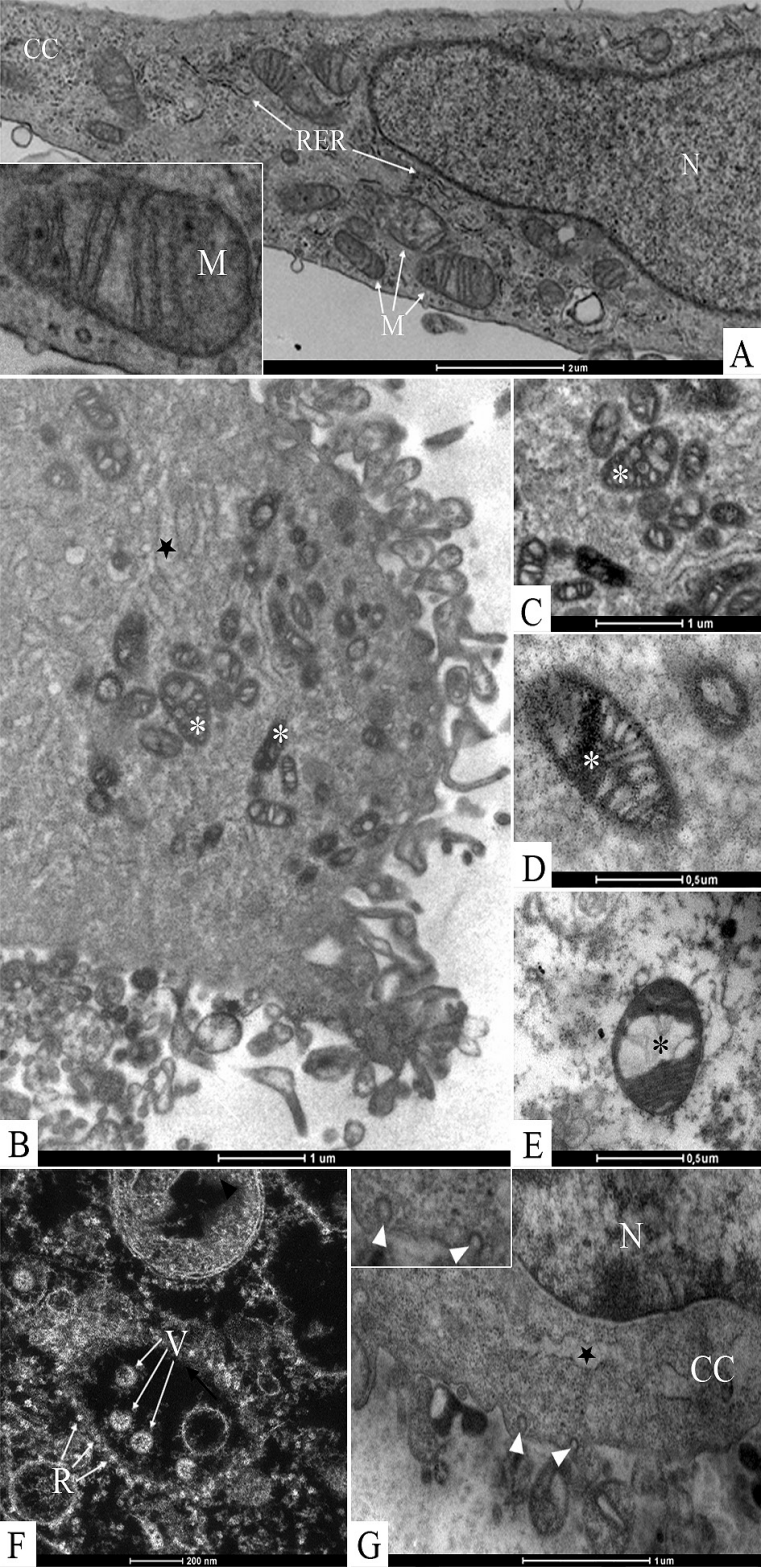
ultrastructural alterations in Vero-E6 72 h post infection (hpi) with severe acute respiratory syndrome (SARS-CoV-2). (A) Uninfected cell presenting no morphological alterations in mitochondria and in rough endoplasmic reticulum cistern (RER). (B-E) Alterations and degeneration of mitochondria (*). (F) Rough endoplasmic reticulum cistern with more electron-dense ribosomes. (B, G) Thickening of the rough endoplasmic reticulum cistern (star). (G) Presence of clathrin-coated vesicles (arrow heads, inset). Nucleus (N), cell cytoplasm (CC).

**Fig. 3:**
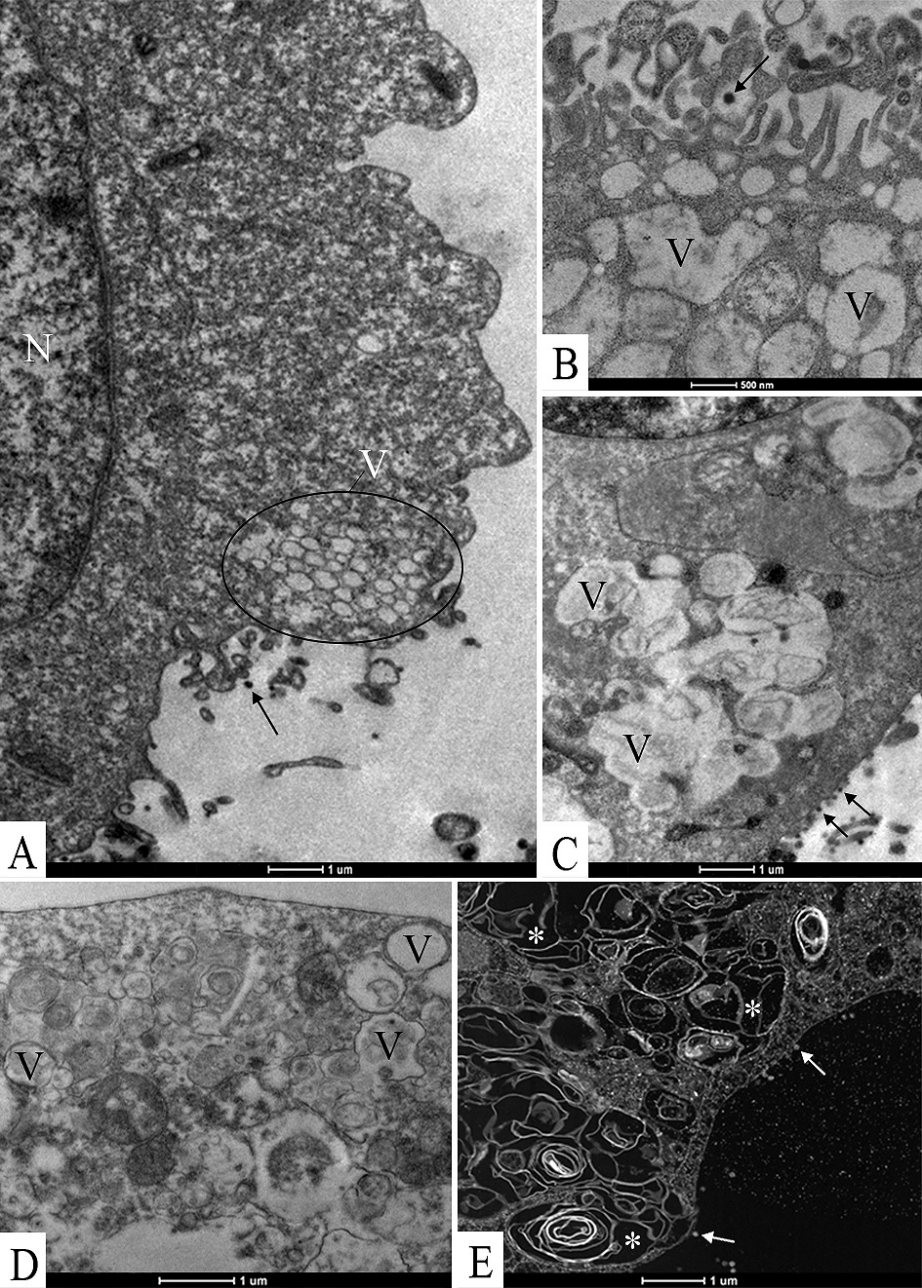
intense smooth vesicle proliferation (V) in Vero-E6 72 h post infection (hpi) with severe acute respiratory syndrome (SARS-CoV-2). (A-D) Vesicle (V) proliferation. Virus particle (B, arrow), nucleus (N). (E) Cell cytoplasm presenting numerous myelin figures (concentric membrane arrays) (*).

**Fig. 4:**
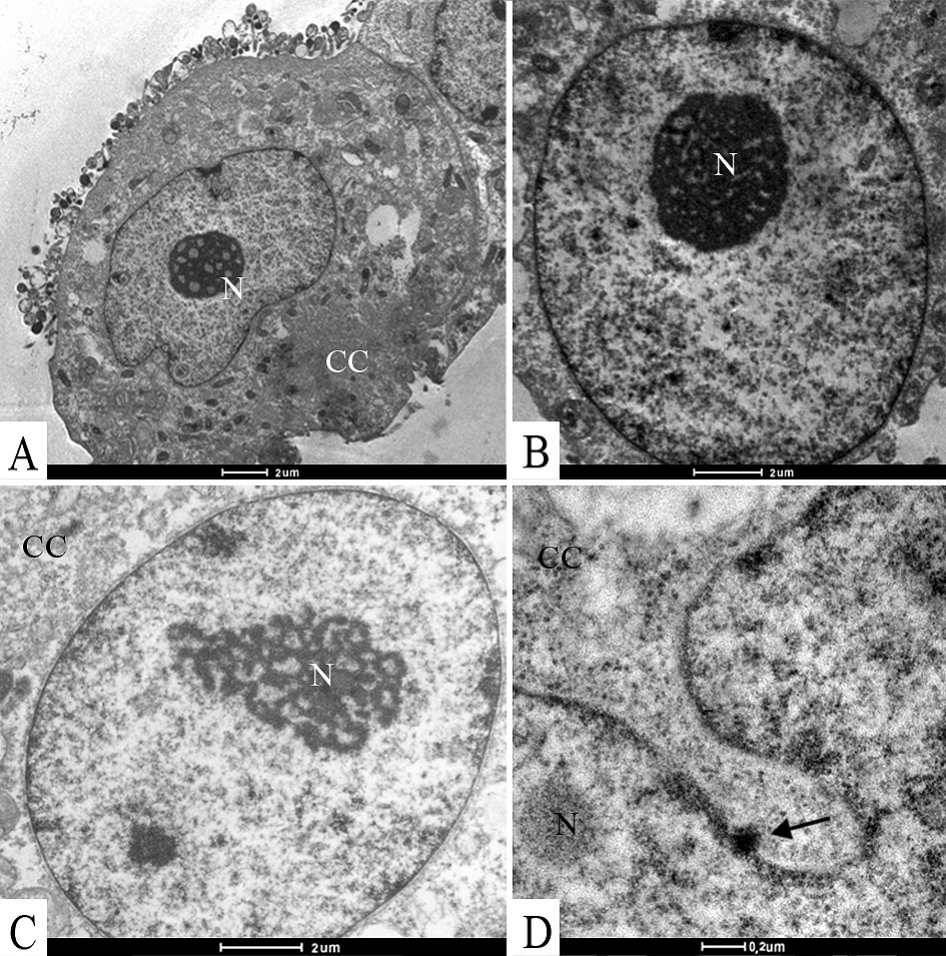
(A-C) alterations of the nucleus (N) chromatin profile in Vero-E6 72 h post infection (hpi) with severe acute respiratory syndrome (SARS-CoV-2). (D) Viral nucleocapsid associated with the nucleus membrane (arrow). Cell cytoplasm (CC).

Ultrastructural analyses of Vero-E6 cells at 72 hpi by transmission electron microscopy showed that the predominant changes associated to SARS-CoV-2 infection were, as follows: cell activation evidencing strong filopodia presence (Fig. 1B-H), alteration and degeneration of mitochondria (Fig. 2B-E), an increased number of more electron-dense ribosomes (Fig. 2F), thickening of the nuclear membrane (data not shown) and RER (Fig. 2B, G), presence of clathrin-coated vesicles (Fig. 2G), smooth vesicle proliferation resulting in a severe vacuolisation of the cells (Fig. 3A-D), numerous myelin figures (Fig. 3E), and chromatin profile change in the nucleus (Fig. 4A-C). As compared with the infected cells, no ultrastructural changes were observed in the uninfected Vero cells (Fig. 1A).

**Fig. 5:**
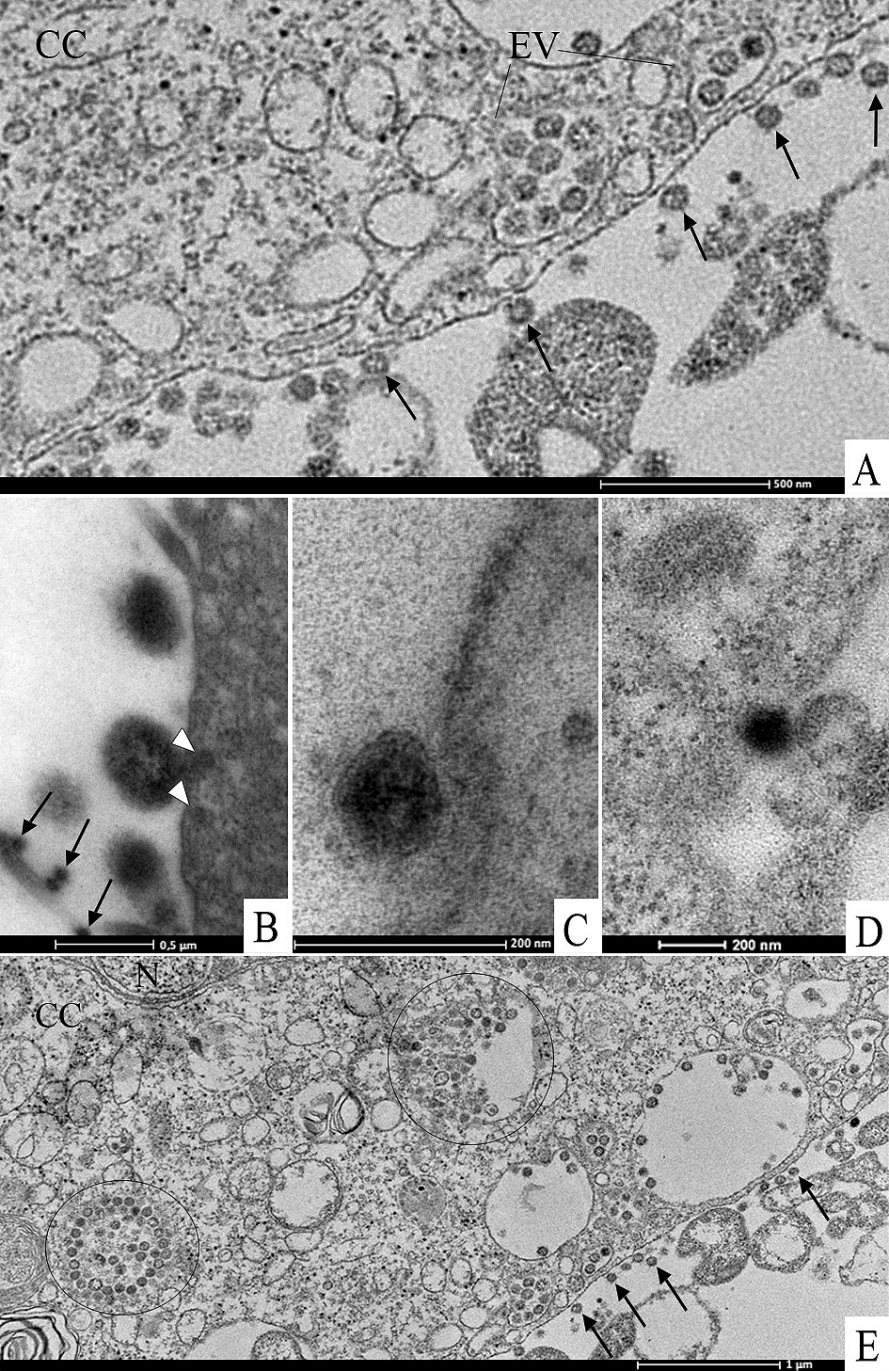
attachment and entry of severe acute respiratory syndrome (SARS-CoV-2) in Vero-E6 cells 72 h post infection (hpi). (A, B, E) Several virus particles (arrows) attached to cell membranes were observed. (C, D) Entry of virus particles into cells by the endocytic pathway and by fusion of virus envelopes with cell membranes (head arrows) (B). (E) Nucleocapsids inside swollen rough endoplasmic reticulum cistern (circles). Cell cytoplasm (CC), endocytic vesicles (EV), nucleus (N).

**Fig. 6:**
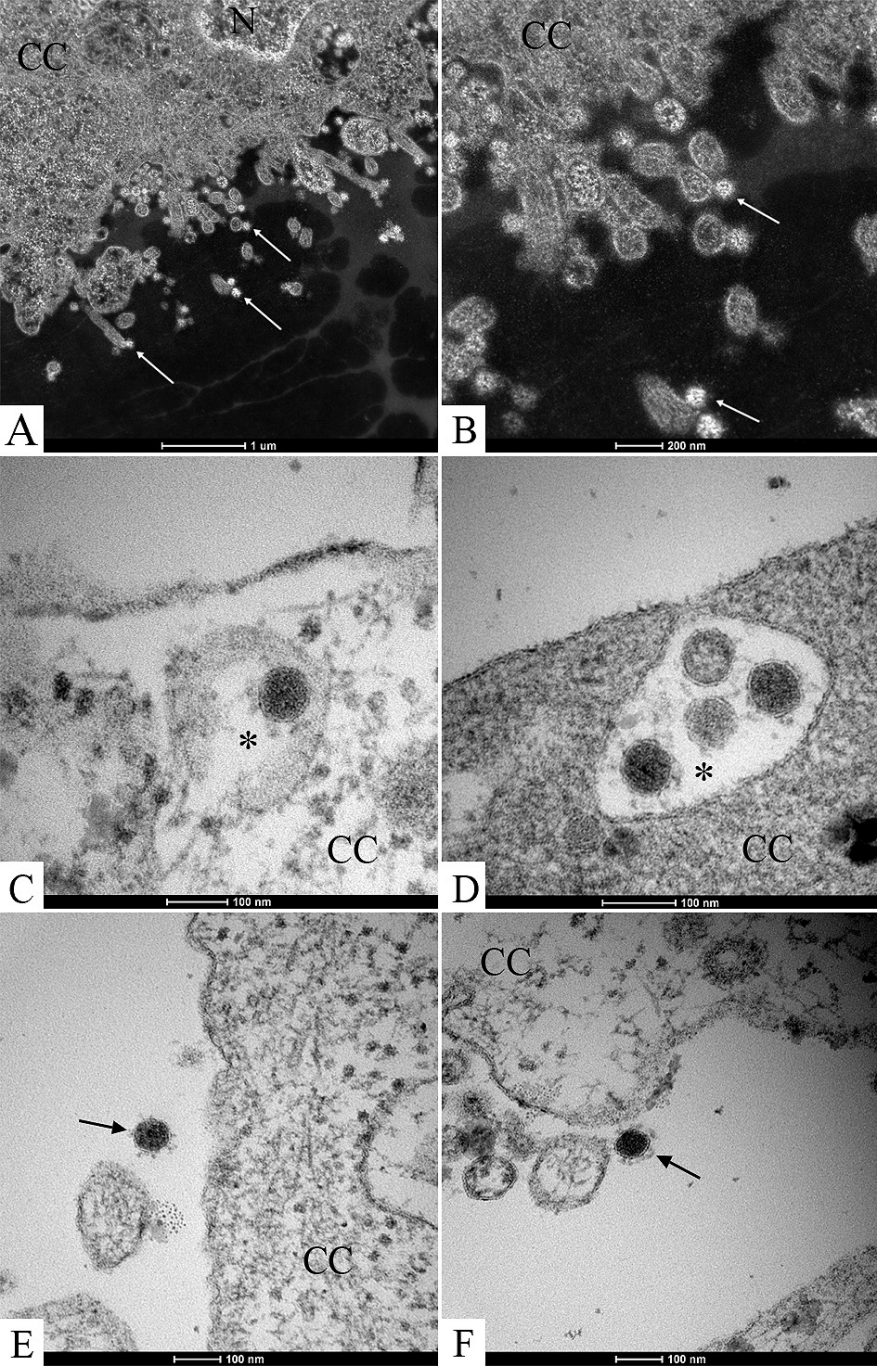
release of severe acute respiratory syndrome (SARS-CoV-2) in Vero-E6 cells 72 h post infection (hpi). (A-E) Virions (arrow) attached to cell filopodia (arrow) and inside smooth vesicles (asterisk) at the periphery of the cell and with your membrane fused with membrane cell were observed. (E-F) Virus particles presenting spherical morphology, displaying spikes, and a diameter between 80 and 100 nm. Cell cytoplasm (CC), nucleus (N).

Virus particles attached to the cell surface (Fig. 5A-B, E) and envelopes fusing with the cell membrane (Fig. 5B) could be observed. Entry of the SARS-CoV-2 virus particles into the cells was observed through fusion of the virus envelope with the cell membrane (Fig. 5B) or by endocytosis (Fig. 5A, C, D). Nucleocapsids were observed inside in the swollen RER (Fig. 5E). The thickened ribosomes attached to the RER membrane were present at few numbers or disappeared completely (Fig. 5E). Nucleocapsids were rarely observed associated with the nucleus membrane (Fig. 4D). Several virions attached to cell filopodia (Fig. 6A-B) and inside smooth vesicles at the periphery of the cell were observed (Fig. 6C). Smooth vesicles with virions inside with membrane fused with membrane cell were observed, too (Fig. 6D). Virus particles presented spherical morphology displaying spikes on its surface (Fig. 6E-F), characteristic of viruses belonging to the *Coronaviridae* family and have a diameter between 80 and 100 nm.

## DISCUSSION

In the present study, using different parameters, we were able to show that Vero-E6 cells are highly permissive to SARS-CoV-2 replication. We observed a logarithmic increase in the number of viral RNA copies in cell culture supernatants, the display of a characteristic cytopathic effect in cell monolayers, and profound alterations in cell ultrastructure, as well as accumulation of viral components and viral particles in different cell compartments and times after infection. These data corroborate those of Park et al.^18^ and Harcourt et al.^19^ that described the susceptibility of this cell lineage for SARS-CoV-2 infection. These authors suggest that Vero-E6 cells might be the best choice for amplification and quantification of the virus. Transmission electron microscopy showed that the predominant changes associated to SARS-CoV-2 infection were cell activation, alteration of mitochondria, thickening of the RER and smooth vesicle proliferation, resulting in a severe vacuolisation of the cells. Our findings are corroborated by the studies carried out by Qinfen et al.^26^ with SARS-CoV, which also observed that, as the infection progresses, the smooth vesicles increased both in number and size. However, while these authors concluded that the smooth vesicles were derived from the Golgi apparatus, our results suggest that they may be related to the RER.

To our knowledge, this is the first ultrastructural characterisation of the morphogenesis of SARS-CoV-2 during viral replication in a cellular model. Our analysis suggests that viral entry into the host cell could occur either by endocytosis or by fusion of the viral envelope with the cell membrane. This is consistent with information from literature, in which we could find studies supporting both mechanisms.^26^ ^,^ ^27^ ^,^ ^28^ Nucleocapsids were observed inside RER cisterns, which presented thickening with a dense electron matrix and gradual loss of ribosomes; more electron-dense ribosomes were also observed. This indicates that part of the SARS-CoV-2 protein synthesis and assembly may occur in the RER. Studies conducted by Zhang et al.^29^ and Qinfen et al.^26^ pointed that the core of SARS-CoV is initially assembled in the RER, where the N protein binds to the genomic RNA and forms the nucleocapsid. The RER gradually loses the ribosomes and swells to become the matrix vesicles that contain the viruses. Qinfen et al.^26^ observed SARS-CoV-like particles in the nucleus of Vero-E6 cells at 48 hpi. Moreover, the nucleic membrane, being in connection with the RER, swelled to form blebs that contained nucleocapsids. These blebs were seen to detach from the nucleic membrane and turn into the virus morphogenesis matrix vesicles.

The presence of virions exclusively inside cytoplasmic smooth vesicles indicates that the particles acquire their envelopes are delivered by budding from the RER directly into smooth vesicles. This process is consistent with those observed with other coronaviruses. Qinfen et al.^26^ demonstrated that the SARS-CoV viral nucleocapsids sprout during morphogenesis from matrix vesicles into these smooth vesicles. In the last step of viral morphogenesis, we observed the virion filled smooth vesicles accumulating in the periphery of the cytoplasm, close to the cell membrane. Subsequent release of viral progeny occurred through the fusion of smooth vesicles with the cell membrane. Similar release mechanisms were described for SARS-CoV, which was also shown to accumulate inside smooth vesicles that move to the cell periphery and eventually fuse with the cell membrane.^26^ The mature SARS-CoV-2 virions had a spherical morphology, a diameter between 80 and 100 nm, and presented the characteristic spikes on the envelopes, which is the signature morphological feature of the coronaviruses. Again, our findings are very consistent with previous *in vitro* studies with SARS-CoV^26^ and SARS-CoV-2, although the last one was reported to present some pleomorphism, and a wider range of virion diameter that varied from 50 to 200 nm.^7^ ^,^ ^13^ ^,^ ^17^ ^,^ ^18^

Further immunomicroscopy and tomography studies are needed to get a better design of the SARS-CoV-2 replication cycle, to better understand the role of the core of SARS-CoV-2 synthesis. The data presented in the present study are important for use in the development of model systems to evaluate therapeutic approaches.

## References

[1] Lin L, Lianfeng Lu, Wei Cao, Taisheng Li (2020). Hypothesis for potential pathogenesis of SARS-CoV-2 infection - a review of immune changes in patients with viral pneumonia. Emerg Microbes Infect.

[2] Sun P, Qie S, Liu Z, Ren J, Li K, Xi J (2020). Clinical characteristics of 50 466 hospitalized patients with 2019-nCoV infection. J Med Virol.

[3] Lai CC, Tzu-Ping S, Wen-ChienKo HJ, Po-RenHsueh (2020). Severe acute respiratory syndrome coronavirus 2 (SARS-CoV-2) and coronavirus disease-2019 (COVID-19) The epidemic and the challenges. Int J Antimicrob Agents.

[4] Lu H, Stratton CW, Tang YW (2020). Outbreak of pneumonia of unknown etiology in Wuhan China the mystery and the miracle. J Med Virol.

[5] Li Q, Xuhua G, Peng W, Xiaoye W, Lei Z, Yeqing T (2020). Early transmission dynamics in Wuhan, China, of novel coronavirus-infected pneumonia. N Engl J Med.

[6] Gorbalenya AE, Susan CB, Ralph SB, Raoul JG, Christian D, Anastasia AG (2020). Severe acute respiratory syndrome-related coronavirus the species and its viruses - a statement of the Coronavirus study. Nat Microbiol.

[7] Chen N, Min Z, Xuan D, Jieming Q, Fengyun G, Yang H (2020). Epidemiological and clinical characteristics of 99 cases of 2019 novel coronavirus pneumonia in Wuhan, China a descriptive study. Lancet.

[8] Huang C, Yeming W, Xingwang L, Lili R, Jianping Z, Yi H, Li Z (2020). Clinical features of patients infected with 2019 novel coronavirus in Wuhan, China. Lancet.

[9] Wang CPW, Horby FG, Hayden GF (2020). A novel coronavirus outbreak of global health concern. Lancet.

[10] Holshue ML, DeBolt C, Scott L, Kathy HL, John W, Hollianne B (2020). First case of 2019 novel coronavirus in the United States. N Engl J Med.

[11] WHO (2020). Coronaviruses disease 2019 (COVID 19) - Situation Report 51. https://www.who.int/docs/default-source/coronaviruse/situation-reports/20200311-sitrep-51-covid-19.pdf?sfvrsn=1ba62e57_10.

[12] Chan JF, Kin-Hang K, Zheng Z, Hin C, Kai-Wang TK, Shuofeng Y (2020). Genomic characterization of the 2019 novel human-pathogenic coronavirus isolated from a patient with atypical pneumonia after visiting Wuhan. Emerg Microbes Infect.

[13] Zhu N, Dingyu Z, Wenling W, Xingwang L, Bo Y, Song J (2020). A novel coronavirus from patients with pneumonia in China, 2019. N Engl J Med.

[14] Jiang S, Du L, Shi Z (2020). An emerging coronavirus causing pneumonia outbreak in Wuhan, China calling for developing therapeutic and prophylactic strategies. Emerg Microbes Infect.

[15] Ren LL, Ye-Ming W, Zhi-Qiang W, Zi-Chun X, Li G, Teng X (2020). Identification of a novel coronavirus causing severe pneumonia in human a descriptive study. Chin Med J(Engl).

[16] Hoffmann M, Kleine-Weber H, Schroeder S, Krüger N, Herrler T, Erichsen S (2020). SARS-CoV-2 cell entry depends on ACE2 and TMPRSS2 and is blocked by clinically proven protease inhibitor. Cell.

[17] Wu C, Yang L, Yueying Y, Peng Z, Wu Z, Yali W (2020). Analysis of therapeutic targets for SARS-CoV-2 and discovery of potential drugs by computational methods. Acta Pharm Sin B.

[18] Park WB, Kwon NJ, Choi SJ, Kang CK, Choe PG, Kim JY (2020). Virus isolation from the first patient with SARS-CoV-2 in Korea. J Korean Med Sci.

[19] Harcourt J, Tamin A, Lu X, Kamili S, Sakthivel SK, Murray J (2020). Severe acute respiratory syndrome Coronavirus 2 from patient with 2019 novel Coronavirus disease, United States. Emerg Infect Dis.

[20] Kim JM, Chung YS, Jo HJ, Lee NJ, Kim MS, Woo SH (2020). Identification of Coronavirus isolated from a patient in Korea with COVID-19. Osong Public Health Res Perspect.

[21] Corman VM, Landt O, Kaiser M, Molenkamp R, Meijer A, Chu DK (2020). Detection of 2019 novel coronavirus (2019-nCoV) by real-time RT-PCR. Euro Surveill.

[22] Szretter KJ, Balish AL, Katz JM (2006). Influenza: propagation, quantification, and storage. Curr Protoc Microbiol.

[23] Barreto-Vieira DF, Barth-Schatzmayr OM, Schatzmayr HG (2010). Modelo animal experimental para o estudo da patogênese dos vírus dengue sorotipos 1 e 2. Interciência.

[24] Barth OM, da Silva MAN, Barreto-Vieira DF (2016). Low impact to fixed cell processing aiming transmission electron microscopy. Mem Inst Oswaldo Cruz.

[25] Reynolds ES (1963). The use of lead citrate at high pH as an electron-opaque stain in electron microscopy. J Cell Biol.

[26] Qinfen Z, Jinming C, Xiaojun H, Huanying Z, Jicheng H, Ling F (2004). The life cycle of SARS coronavirus in Vero E6 cells. J Med Virol.

[27] Song Z, Xu Y, Bao L, Zhang L, Yu P, Qu Y (2019). From SARS to MERS, thrusting coronaviruses into the spotlight. Viruses.

[28] Ng ML, Tan SH, See EE, Ooi EE, Ling AE (2003). Early events of SARS coronavirus infection in Vero cells. J Med Virol.

[29] Zhang Q, Cui JM, Huang XJ, Lin W, Tan DY, Xu JW (2003). Morphology and morphogenesis of severe acute respiratory syndrome (SARS) - associated virus. Sheng Wu Hua Xue Yu Sheng Wu Wu Li Xue Bao (Shanghai).

